# Interleukin-32*γ* in the Control of Acute Experimental Chagas Disease

**DOI:** 10.1155/2022/7070301

**Published:** 2022-01-20

**Authors:** Yarlla L. L. Braga, José R. C. Neto, Arthur W. F. Costa, Muriel V. T. Silva, Marcos V. Silva, Mara R. N. Celes, Milton A. P. Oliveira, Leo A. B. Joosten, Fátima Ribeiro-Dias, Rodrigo S. Gomes, Juliana R. Machado

**Affiliations:** ^1^Instituto de Patologia Tropical e Saúde Pública, Universidade Federal de Goiás, Goiânia, GO, Brazil; ^2^Departamento de Microbiologia, Bioquímica e Imunologia, Instituto de Ciências Biológicas e Naturais, Universidade Federal do Triângulo Mineiro, Uberaba, MG, Brazil; ^3^Department of Internal Medicine and Radboud Center of Infectious Diseases (RCI), Radboud University Medical Center, Nijmegen, Netherlands; ^4^Departamento de Patologia, Genética e Evolução, Instituto de Ciências Biológicas e Naturais, Universidade Federal do Triângulo Mineiro, Uberaba, MG, Brazil

## Abstract

Chagas disease (CD) is an important parasitic disease caused by *Trypanosoma cruzi*. Interleukin-32 (IL-32) plays an important role in inflammation and in the development of Th1/Th17 acquired immune responses. We evaluated the influence of IL-32*γ* on the immune response profile, pathogenesis of myocarditis in acute experimental CD, and control of the disease. For this, C57BL/6 wild-type (WT) and IL-32*γ*Tg mice were infected subcutaneously with 1,000 forms of Colombian strain of *T. cruzi*. In the histopathological analyzes, *T. cruzi* nests, myocarditis, and collagen were quantified in cardiac tissue. Cytokine productions (IL-32, IFN-*γ*, TNF-*α*, IL-10, and IL-17) were measured in cardiac homogenate by ELISA. The IL-32*γ*Tg mice showed a better control of parasitemia and *T. cruzi* nests in the heart than WT mice. Infected-WT and -IL-32*γ*Tg mice showed similar levels of IFN-*γ*, TNF-*α*, and IL-17, but IL-10 was significantly higher expressed in IL-32*γ*Tg than in WT mice. The cytokine profile found in IL-32*γ*Tg animals contributed to body weight maintenance, parasitemia control, and survival. Our results indicate that the presence of human IL-32*γ* in mice infected with the Colombian strain of *T. cruzi* is important for infection control during the acute phase of Chagas disease.

## 1. Introduction

Chagas disease (CD), also called American Tryponosomiasis, is a parasitic disease resulting from infection by *Trypanosoma cruzi*. It is estimated that there are about 8 million people infected worldwide, with a high prevalence in Latin America, where CD is endemic [1]. For individuals who develop clinical manifestations, cardiomyopathy is one of the main pathological processes of CD and affects 20-30% of infected individuals [2]. Transmission in humans occurs mainly via the vector route, but infection can occur through blood transfusions, organ transplants, laboratory accidents, congenital, and oral transmission [1].

The *T. cruzi*, in its metacyclic trypomastigote form, is capable of infecting several cell types such as macrophages, fibroblasts, epithelial cells, among other cells [3, 4]. Infection by *T. cruzi* culminates in the simultaneous activation of defense mechanisms of the innate and adaptive immunity [5]. At the beginning of the infection, macrophages secrete interleukin- (IL-) 12 (IL-12), which, in turn, favors Th1 response profile [6–9]. The production of Th1 cytokines such as interferon gamma (IFN-*γ*) and tumor necrosis factor *α* (TNF-*α*) will drive a microbicidal mechanism against *T. cruzi*, inducing the synthesis of nitric oxide (NO) by macrophages [6–9]. As a control mechanism of the immune response, cytokines with a regulatory profile such as IL-10 and transforming growth factor *β* (TGF-*β*) are released and inhibit the proinflammatory effects [10, 11].

The IL-32 is an intracellular cytokine described in 2005 [12], produced by immune and nonimmune cells, and has nine isoforms generated by alternative processing of IL-32*γ* mRNA, the most biologically active isoform [13, 14]. IL-32*γ* is characterized as a proinflammatory cytokine, which induces TNF-*α*, IL-6, IL-8, and IL-1*β* [12, 15]. However, it has been shown that IL-32*β* is able to induce IL-10, a regulatory cytokine, thus highlighting different regulatory effects for IL-32 isoforms [16]. Expression of IL-32 has been associated with the severity of inflammation in rheumatoid arthritis and the control of infection with *Mycobacterium tuberculosis* and *M. bovis* as well as the pathogenesis of chronic hepatitis B [17–21].

Mouse cells, despite responding to IL-32 stimulation, do not produce this cytokine. Thus, C57BL/6 transgenic mouse expressing human IL-32*γ* gene (IL-32*γ*Tg mice) has been developed and used as an experimental model for different infectious diseases [19, 22–24]. Our group recently demonstrated that IL-32*γ* in transgenic mice infected with *Leishmania infantum* was able to induce a Th1/IFN-*γ*/TNF-*α* and Th17/IL-17 profiles, which lead to increase of NO production to protect against the infection [23]. Previously, we have shown that IL-32*γ* was highly expressed in lesions of patients with American Tegumentary Leishmaniasis caused by *Leishmania* (*Viannia*) sp. or *L. amazonensis* [25]. Since these studies showed that IL-32 can be induced by protozoa and modulate the immune responses, we investigated whether IL-32*γ* has a protective and/or immunopathogenic effect during the acute experimental murine CD, using the Colombian strain, highly myotropic, and IL-32*γ*Tg mice [26].

## 2. Methodology

### 2.1. Infection

The animals used in this study were bred and maintained in animal facility of the Institute of Tropical Pathology and Public Health of the Federal University of Goiás. Male C57BL/6 (wild-type (WT)) and C57BL/6 transgenic for human IL-32*γ* (IL-32*γ*Tg) were 8-10 weeks of age (22-27 g). They were grouped as WT or IL-32*γ*Tg noninfected (controls) and infected mice, which were infected with the Colombian strain of *T. cruzi*. For infection, 1,000 trypomastigote forms were inoculated subcutaneously in the ventral region of each animal. During the experiment period, the animals were supervised daily. Euthanasia occurred on the 28th day of infection to reach the acute phase. Besides that, to evaluate IL-32 levels, IL-32*γ*Tg controls and infected animals were euthanized on the 14th day of infection. Euthanasia was done by cervical dislocation after confirmation of the anesthetic status induced by 50 mg/kg of xylazine hydrochloride intraperitoneally. Necropsy was performed by ventral incision of the thoracic and abdominal cavities, thus collecting the spleen and the heart for studies. The Research Project of this work is approved by CEUA/UFG, under protocol No. 042/16.

### 2.2. Assessment of Parasite Load, Survival, and Body Weight

The parasitemia of infected mice was evaluated at an interval of three days, starting on the third day of infection and continuing until the 27th day, using the method of Brener (1968). The mice were also weighed on the day of infection (day 0) and on the day of euthanasia (day 28). During this period, animals were supervised daily for survival verification.

### 2.3. Histological Analysis

To quantify the inflammatory infiltrate and amastigote nests, hematoxylin-eosin (HE) stained heart fragment slides were used. Tissue parasitism was evaluated on three slides with serial sections. The analyses were performed from the capture of 20 random fields in the ventricles photographed at 400x magnification by means of a digital camera coupled to a microscope (Leica QWin) and evaluated in the TMARKER program for quantification. Thus, the results were expressed in density of infiltrated cells and number of nests/mm^2^. The morphometric evaluation of the fibrous connective tissue was performed by Picrosirius staining. Ten random fields in the ventricles were captured and quantified at 400x magnification, using a polarized light microscope. The quantification of collagen was performed using the Axion vision software (ZEISS), and the results were expressed as a percentage of collagen/animal.

### 2.4. Cardiac Tissue Homogenate

On the 28th day of infection, the heart and spleen were collected and quickly washed in saline solution, dried on filter paper, and weighed on a precision scale. The organs were subsequently subjected to a cross-section, in which half were destined for histopathological analysis, and the other was stored in a freezer at -80°C in a PBS solution containing a complete™ protease inhibitor (Sigma, USA). Subsequently, the frozen material was homogenized with the aid of a homogenizer (Dremel, USA), and the homogenate was centrifuged at 13000 g for 30 minutes at 4°C. The supernatant was collected and stored for determination of IL-32 levels and total proteins.

### 2.5. Cytokine Quantification

The evaluation of IFN-*γ*, IL-10, IL-32, and TNF-*α* levels in cardiac and spleen homogenates was performed by a sandwich enzyme immunoassay (ELISA) method, according to the manufacturers' protocol (R&D System for IL-32 and BDEIAOptEIA™ for other cytokines). The concentration of cytokines in the samples was determined by regression analysis of absorbance (BIORAD 2550 READER EIA, USA) obtained from the respective standards. The measure was normalized by the total protein concentration in the sample, and the results were expressed in pg/mg.

### 2.6. Statistical Analysis

Statistical analysis was performed using the GraphPad Prism 6.0 program (GraphPad Software—USA). To compare two groups, the unpaired *t*-test was used for data with normal distribution and the Mann–Whitney test for data with nonnormal distribution. For comparison of more groups, the one-way ANOVA test was used when the data had a normal distribution and the Kruskal-Wallis test for nonnormal data. For comparison analysis of two groups at different times, the two-way ANOVA test was used. Multiple *t*-tests were used for more than two groups. Survival analysis were evaluated by the Log-Rank test. The results were considered statistically significant when *p* < 0.05.

## 3. Results

### 3.1. IL-32*γ* Controls Parasitemia and Protects Mice in the Acute Phase of Chagas Disease

**Figure 1:**
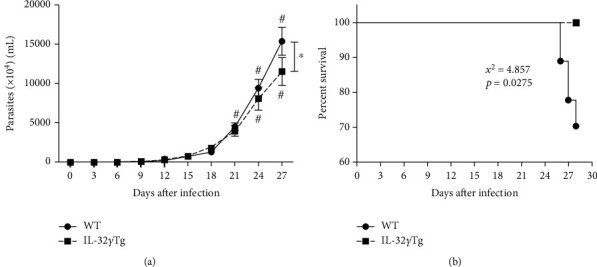
Interleukin-32*γ* controls parasitemia and protects mice in the acute phase of Chagas disease. WT and IL-32*γ*Tg mice were subcutaneously infected with 1,000 blood trypomastigote forms of *T. cruzi* Colombian strain. (a) Parasitemia was determined by counting the number of trypomastigotes in blood collected from the caudal vein. #*p* < 0.05 (differences within the same group, comparing first day with detectable parasitemia vs. the time point observed) and ^∗^*p* < 0.05 (WT vs. IL-32*γ*Tg mice). Significant statistical differences at *p* < 0.05, two-way ANOVA with Bonferroni post hoc test. (b) Percent survival of infected IL-32*γ*Tg and WT mice, *p* = 0.0275, Log-Rank test.

Parasitemia was evaluated in WT- and IL-32*γ*Tg-infected animals at three-day intervals, starting on the 3rd day until the 27th day of infection. Parasitemia in the WT and IL-32*γ*Tg mice were predominantly detectable from the 9th day of infection. In the WT mice, parasitemia was significantly increased from the 21st day of infection while in the IL-32*γ*Tg mice, it was from the 24th day of infection. We observed that, on the 27th day of infection, the WT group had a higher number of blood trypomastigotes than the IL-32*γ*Tg mice (Figure 1(a)). Survival rate was evaluated in WT- and IL-32*γ*Tg-infected animals during 28 days. The WT mice started to die 26 days postinfection, showing 68% survival (17/25 animals) while IL-32*γ*Tg mice presented 100% survival (14/14 animals; *χ*^2^ = 4.857, *p* = 0.0275; Figure 1(b)).

**Figure 2:**
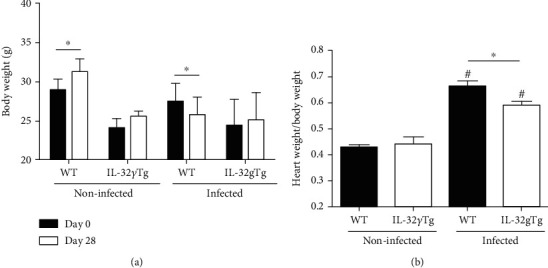
Body and heart weight of WT and IL-32*γ*Tg mice noninfected or infected with the Colombian strain of *T. cruzi* in the acute phase of experimental Chagas disease. (a) Body weight evaluated on day 0 and day 28 of infection. ^∗^*p* < 0.05 (day 0 vs. day 28, two-way ANOVA test). (b) Heart weight and body weight ratio of noninfected and infected WT and IL-32*γ*Tg mice (%). #*p* < 0.05 (differences within the same animal group) and ^∗^*p* < 0.05 (WT vs. IL-32*γ*Tg mice; two-way ANOVA test with Bonferroni post hoc test).

To assess the clinical course of infection, uninfected and infected mice were weighed on the day of infection (day 0) and on the day of euthanasia (day 28). During this period, the noninfected WT mice showed a significant increase in body weight while the infected WT mice showed a significant weight reduction. No changes were observed between IL-32*γ*Tg animals infected or noninfected in the two evaluation periods (Figure 2(a)). The relative weight (organ/body) of mouse hearts was also analyzed on the 28th day of infection. The hearts of infected WT showed a significant increase in relative weight when compared to the hearts of noninfected WT mice. Similarly, infection in IL-32*γ*Tg mice also induced a significant increase in the relative weight of the heart. However, infected-WT mice presented a higher relative heart weight than infected-IL-32*γ*Tg mice (Figure 2(b)).

### 3.2. IL-32*γ* Does Not Change Inflammatory Infiltrate and Cardiac Collagen Deposition, but Controls Tissue Parasitism during Experimental Chagas Disease

**Figure 3:**
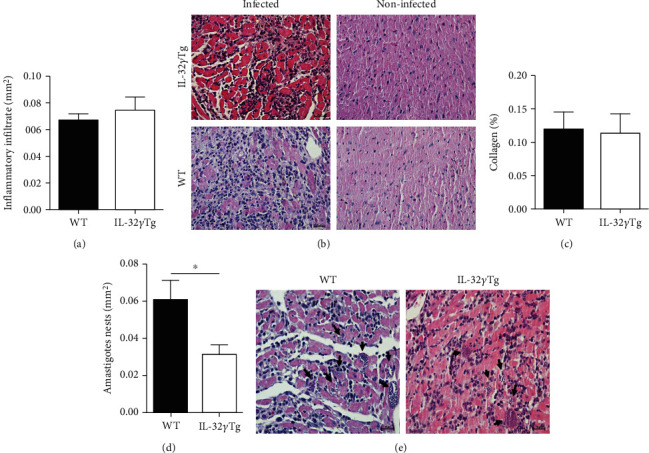
Morphological analysis of the heart of WT and IL-32*γ*Tg mice noninfected or infected with the Colombian strain of *T. cruzi* in the acute phase of experimental Chagas disease. (a) Density of cardiac inflammatory infiltrate in infected WT and IL-32*γ*Tg mice. (b) Inflammatory infiltrate in the hearts of infected WT and IL-32*γ*Tg mice (HE, 40x). (c) Percentage of collagen deposited in the heart. (d) Density and (e) histological sections of the amastigote nests in cardiac tissue of infected WT and IL-32*γ*Tg mice (HE, 40x). ^∗^*p* < 0.05 (Student's *t*-test).

The analyses of the cardiac inflammatory process were performed in infected WT and IL-32*γ*Tg mice 28 days after infection. It was not possible to observe significant differences in the inflammatory infiltrate between the groups (Figures 3(a) and 3(b)). Analysis of collagen deposition was performed using digital morphometry on PS-stained heart slides. In this analysis, no significant difference was observed between the groups (Figure 3(c)).

Tissue parasitism was quantified in three serial sections of cardiac tissue from infected mice. Nests of amastigotes distributed throughout the right and left ventricles were observed. IL-32*γ*Tg mice showed a significant reduction in the density of amastigotes nests when compared to WT mice on the 28th day of infection (Figures 3(d) and 3(e)).

### 3.3. IL-32*γ* Induces High Production of IL-10 in Cardiac Tissue during Experimental Chagas Disease

**Figure 4:**
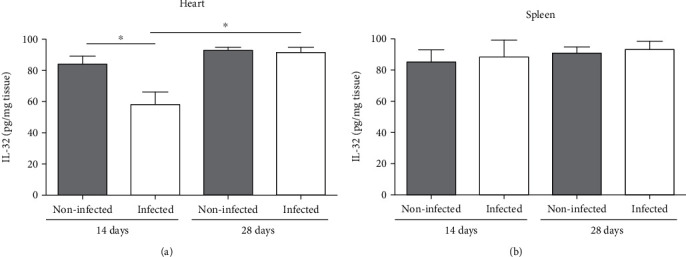
Levels of IL-32 in the (a) heart and (b) spleen of noninfected or infected IL-32*γ*Tg mice. Mice were infected with Colombian strain of *T. cruzi*, and IL-32 was measured in heart tissue during the acute phase of experimental Chagas disease. ^∗^*p* < 0.05 (two-way ANOVA test with Bonferroni post hoc test).

The intracellular cytokine IL-32 was measured in the homogenate of the heart and spleen of noninfected and infected IL-32*γ*Tg mice. Infection with *T. cruzi* reduced the amount of IL-32 in the cardiac tissue after 15 days, but after 28 days, the levels were recovered to the levels of noninfected mice (Figure 4(a)). In addition, we did not observe effects of infection on IL-32 levels in the spleen of IL-32*γ*Tg mice at any time evaluated (Figure 4(b)).

**Figure 5:**
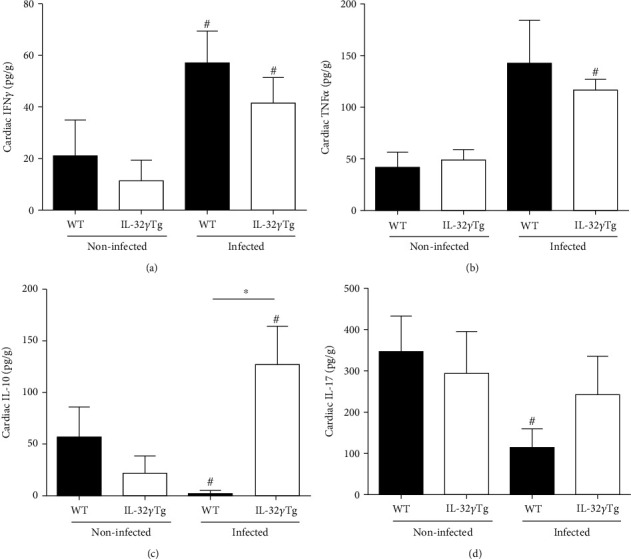
Effects of IL-32*γ* on cytokine production in the hearts of mice infected with the Colombian strain of *T. cruzi* during acute phase of experimental Chagas disease. Quantification levels of (a) IFN*γ*, (b) TNF-*α*, (c) IL-10, and (d) IL-17 (pg/mg) in cardiac homogenates by ELISA. ^∗^*p* < 0.05 (WT vs. IL-32*γ*Tg mice), #*p* < 0.05 (noninfected vs. infected within the same group of animals, two-way ANOVA test with Bonferroni post hoc test).

The cytokines IFN-*γ*, TNF-*α*, IL-10, and IL-17 were also measured in the homogenate of the heart after 28 days of infection. There were no significant differences in the levels of all cytokines in noninfected WT or IL-32*γ*Tg mice (Figure 5). The infection induced a significant increase of IFN-*γ* levels in both groups WT and IL-32*γ*Tg mice, without difference between these groups (Figure 5(a)). Although in WT mice, the TNF-*α* production was increased after infection statistical significance was not achieved; in IL-32*γ*Tg mice, there was significant increase of TNF-*α* production (Figure 5(b)). On the other hand, *T. cruzi* infection decreased the basal levels of IL-17 in WT mice whereas in IL-32*γ*Tg mice the levels were maintained (Figure 5(d)). The presence of IL-32*γ* during the infection increased the cardiac levels of IL-10, which were higher in IL-32*γ*Tg than in WT mice. In WT mice, the levels of this cytokine decreased after *T. cruzi* infection (Figure 5(c)).

## 4. Discussion

In the present study, we evaluated the role of IL-32*γ* in the immune response and cardiac alterations resulting from experimental infection with the Colombian strain of *T. cruzi*, during the acute experimental phase. The presence of IL-32 was not able to induce an increase in basal cytokines in the noninfected control group. This is demonstrated by the similarity in the levels produced in the heart when compared to the noninfected WT group. Thus, the participation of Il-32, in fact, occurs from the establishment of *T. cruzi* infection, thus indicating that IL-32*γ*Tg mice are suitable for use in this study. While WT animals showed weight loss after infection, IL-32*γ*Tg animals did not show a change in body weight. *T. cruzi* infection promotes weight loss in different experimental models [27, 28]. It is known that proinflammatory cytokines such as TNF-*α*, IL-6, IL-1, and IFN-*γ* are involved in cachexia mechanisms found in experimental animals [29, 30]. In this study, we could observe that IL-10, a regulatory cytokine, is being highly produced in the cardiac homogenate of IL-32*γ*Tg mice. Then, we believe that this cytokine may be regulating the immune response and thus justifying the maintenance of weight in these animals.

In the present experiments, mice infected with *T. cruzi* had predominantly detectable parasitemia from the 9th day of infection. In fact, it is known that during the first seven days of infection, the parasitemia in experimental models is not detected, and this occurs because the parasite proliferation is still restricted to the inoculum region [28]. On the 27th day, it was possible to notice that IL-32*γ*Tg animals presented a significantly lower parasitemia than WT animals, suggesting that this cytokine is involved in the control of blood trypomastigotes. It is known that IL-32*γ* is able to induce Th1 cell differentiation, which produce IFN-*γ* [14]. In fact, in infection with another trypanosomatide, *L. infantum*, it was observed that IL-32*γ* was able to induce a Th1/IFN-*γ* profile, leading to a protective effect against infection [23]. IFN-*γ* is one of the cytokines most closely involved in resistance to infection by *T. cruzi* [7, 10, 11], participating in the inhibition of intracellular replication of the parasites, activation/maintenance of the Th1 response, and production of antibodies [31, 32]. Our data suggest that IL-32*γ* is contributing to the control of the parasitemia by mechanisms that are dependent on IFN-*γ* but also by IFN*γ*-independent pathways.

In addition to IFN-*γ*, TNF-*α* is an essential cytokine for the control of *T. cruzi* in the acute phase of experimental infection. TNF-*α* is part of the group of Th1 profile cytokines, which act synergistically with IFN-*γ* in macrophages to increase NO production [6], which, in turn, destroys the parasites at the beginning of the infection. In the study in focus, an increase cardiac IFN-*γ* was observed in both groups infected when compared with respective controls, although only in IL-32*γ*Tg mice *T. cruzi* infection induced significant high levels of TNF-*α*, which may be related to the better control of infection observed in these animals. Considering that IL-32*γ*Tg infected animals produce high levels of IL-10 concomitantly with TNF-*α* in the heart, it can be suggested that TNF-*α* and IFN-*γ* are related to the decrease in cardiac parasitism in this group while, at the same time, the high production of IL-10 is related to the regulation of the immune response and a counterbalance of the toxic action of TNF-*α* in IL-32*γ*Tg mice, which results in the protection of the heart.

Collagen analysis showed no differences between control and infected groups. Such results can be explained by the fact that the evaluation was carried out in the acute phase of the infection. Fibrosis is the tissue repair that causes tissue destruction characteristic of CD. Thus, it would be a later response, which is not observed in the early stages of the disease [2, 33, 34]. Furthermore, there are no reports on the role of IL-32*γ* in inducing fibrosis. To evaluate the fibrosis in experimental CD and the expression of IL-32*γ* is extremely relevant as it can provide information that contributes to the understanding of the pathogenesis of the disease.

Here, it was possible to observe amastigote nests distributed throughout the ventricle. IL-32*γ*Tg infected mice had a significantly lower amastigote nest density than infected WT animals. The Colombian strain has a clear myotropism [26]. It is known that during acute phase of CD infection with the Colombian strain there is an intense cardiac parasitism in mice [35, 36]. Our data suggest that the smaller number of amastigote nests found in the hearts of IL-32*γ*Tg animals is due to the control of circulating parasites by IL-32*γ*-mediated mechanisms, added to the effects of TNF-*α* and IFN-*γ* in situ.

The Colombian strain in C57BL/6 animals is able to induce an intense cell infiltrate in the myocardium [33]. The effects of IL-32*γ* on inflammatory processes in the heart are still unknown; however, in an experimental rheumatoid arthritis model, this cytokine induced an intense cell infiltrate in the joints of C57BL/6 animals [17]. We did not observe differences in the density of the inflammatory infiltrate in the heart of the IL-32*γ*Tg and WT infected groups. Although levels of myocarditis in our model were not different, tissue production of IL-10 was higher in the IL-32*γ*Tg group of animals. It is known that alternative splicing of IL-32*γ* mRNA can generate other isoforms of this cytokine, including IL-32*β* [14, 37]. IL-32*β* is able to induce IL-10, an anti-inflammatory cytokine [16] that inhibits the effects of IFN-*γ* and, consequently, the production of NO by macrophages [11]. Our results suggest that the immune response profile in IL-32*γ*Tg mice, after 28 days of infection, became more regulated due to the high production of IL-10 observed in the heart, and this was due to a possible induction of IL-32*β*. Then, this would be the reason by why the hearts of IL-32*γ*Tg mice present a lower relative weight than that of the infected-WT animals. Levels of IL-10 in WT-infected animals when compared to their controls showed a significant decrease. Thus, the characteristic alterations of an inflammatory process, such as edema and congestion, present in acute myocarditis [38] would be more evident in the hearts of infected-WT animals causing their increased weights.

In IL-32*γ*Tg mice, the production of IL-10 was associated with less severity of colitis process [22]. In contrast, in *M. tuberculosis* infection, the increased production of IL-10 was associated with a decreased protective response against these bacteria in IL-32*γ*Tg mice [19]. In IL-32*γ*Tg, infection with *T. cruzi* significantly decreased IL-32 production in situ at 14 days postinfection. The levels of this cytokine returned to basal levels 28 days after infection. The *T. cruzi* evasion mechanisms [39, 40] may be inducing a decrease in IL-32*γ* production on the 14th day of infection, at the beginning of the infectious process, in an attempt to establish the parasitism. However, the data from this study suggest that IL-32 can be induced later in infection, which contributes to reduce clinical changes of CD, and to eliminate, at least in part, the parasites, reducing the parasitemia. Our data suggest that the effects of IL-32 in *T. cruzi* infection could be even more pronounced if the parasite did not reduce the production of this cytokine at the beginning of the infection. It is crucial to further investigate whether the escape mechanisms of *T. cruzi* can involve inhibition of IL-32 production in human cells.

We have observed that *T. cruzi* infection led to a drop in the IL-17 levels in the hearts of WT mice whereas the production of this cytokine was maintained in the IL-32*γ*Tg mice. It is known that IL-17 and IL-32 are able to induce each other [41] and share common targets in signaling pathway impacting TNF-*α* and IL-1*β*-mediated cellular responses [42]. We have demonstrated that during the infection of IL-32*γ*Tg mice with *L. infantum* IL-32*γ* stimulated IL-17, which played an essential role in NO production [23]. In other diseases, IL-32 has contributed to a Th17 response profile [41–44]. It is known that IL-17 plays an important role in the control of infection with *T. cruzi.* Mice infected with Talahuén strain and treated with IL-17 neutralizing antibodies showed an increased parasitemia and mortality, besides reduced production of inflammatory cytokines [45]. However, there is controversy regarding the role of IL-17 in *T. cruzi* infection. After neutralization of IL-17 in mice infected with the Y strain, increased mortality, myocarditis with high production of inflammatory cytokines, and low tissue parasitism were observed [46]. Thus, it was suggested that IL-17 would be inducing negative feedback on cytokines such as IFN-*γ* and TNF-*α* [5, 42]. It has been shown that patients who present a mild heart disease or are in the indeterminate phase of CD produce high levels of IL-17 and IL-10 whereas patients with severe heart disease show high levels of IFN-*γ* and TNF-*α* [47–49]. The data from the present study suggest that IL-32*γ* in the heart lead to maintenance of IL-17 levels, producing an infection control and, together with IL-10, regulation of the immune response in order to avoid an intense inflammatory response and consequently cardiac damage. However, the real role of IL-17 in the outcome of CD still needs to be better elucidated.

In infected animals, survival rates of 100% in IL-32*γ*Tg-infected mice and 68% in WT mice were observed. All the mechanisms discussed above were determining factors for this result. The data suggest that the presence of IL-32*γ* in mice infected with the Colombian strain of *T. cruzi* is important for the control of parasitemia and cardiac parasitism as well as for the maintenance of the animals' weight and survival during the acute phase of the infection. The increased expression of IL-10 in situ in IL-32*γ*Tg mice may be one of the relevant mechanisms involved in the control and immunopathogenesis of the experimental infection against this protozoan. To understand the role of IL-32 in CD is crucial to reveal the landscape of *T. cruzi* control and the immunopathogenesis in the disease.

## Data Availability

The original contributions presented in the study are included in the article. Further inquiries can be directed to the corresponding authors.
